# EEG Phase Synchronization in Persons With Depression Subjected to Transcranial Magnetic Stimulation

**DOI:** 10.3389/fnins.2018.01037

**Published:** 2019-01-14

**Authors:** Urszula Zuchowicz, Agata Wozniak-Kwasniewska, David Szekely, Elzbieta Olejarczyk, Olivier David

**Affiliations:** ^1^Department of Automatics and Biomedical Engineering, AGH University of Science and Technology, Cracow, Poland; ^2^Inserm, U1216, Grenoble, France; ^3^Univ. Grenoble Alpes, Grenoble Institut des Neurosciences, Grenoble, France; ^4^Centre Hospitalier Univ. Grenoble Alpes, Service de Psychiatrie, Grenoble, France; ^5^Nalecz Institute of Biocybernetics and Biomedical Engineering, Polish Academy of Sciences, Warsaw, Poland

**Keywords:** EEG, repetitive transcranial magnetic stimulation, brain connectivity, indices based on graph theory, Phase Locking Value, major depression disorder, bipolar disorder, depression

## Abstract

**Aim:** The main objective of this work was to study the impact of repetitive Transcranial Magnetic Stimulation (rTMS) treatment on brain activity in 8 patients with major depressive disorder (MDD) and 10 patients with bipolar disorder (BP). Changes due to rTMS stimulation of the left dorsolateral prefrontal cortex (DLPFC) were investigated considering separately responders and non-responders to therapy in each of both groups. The aim of the research is to determine whether non-responders differ from responders suffered from both diseases, as well as if any change occurred due to rTMS across consecutive rTMS sessions.

**Methods:** The graph-theory-based connectivity analysis of non-linearity measure of phase interdependencies—Phase Locking Value (PLV)—was examined from EEG data. The approximately 15-min EEG recordings from each of participants were recorded before and after 1st, 10th, and 20th session, respectively. PLV calculated from data was analyzed using principal graph theory indices (strength and degree) within five physiological frequency bands and in individual channels separately. The impact of rTMS on the EEG connectivity in every group of patients evaluated by PLV was assessed.

**Results:** Each of four groups reacted differently to rTMS treatment. The strength and degree of PLV increased in gamma band in both groups of responders. Moreover, an increase of indices in beta band for BP-responders was observed. While, in MDD-non-responders the indices decreased in gamma band and increased in beta band. Moreover, the index strength was lower in alpha band for BP- non-responders. The rTMS stimulation caused topographically specific changes, i.e., the increase of the activity in the left DLPFC as well as in other brain regions such as right parieto-occipital areas.

**Conclusions:** The analysis of PLV allowed for evaluation of the rTMS impact on the EEG activity in each group of patients. The changes of PLV under stimulation might be a good indicator of response to depression treatment permitting to improve the effectiveness of therapy.

## Introduction

Major depressive disorder (MDD) is one of the most common mental disorders in the world and leads to a variety of emotional and physical problems such us overwhelming feeling of sadness and isolation, frequent anxiety and irritation, diminished self-esteem and reduction in attention or concentration (American Psychiatric Association (APA), 2013). According to recent estimation about 300 million people suffer from depression episodes, and, as a result, about 800,000 patients commit suicide (World Health Organization (WHO), 1992, 2017; National Institutes of Health (NIH), 2007). In psychiatry, depression classification includes also the disease entity termed bipolar affective disorder. It is marked by depressive and manic episodes which occur in repeated periods. The origin of MDD is associated with functional deficits, abnormal structures of brain and defective activity of neurotransmitters. Numerous researches have demonstrated the differences in physiologic brain mechanisms between healthy people and patients with depression. The pathophysiology is observed not only in abnormal changes in structures in brain but also in bioelectrical activity of neurons. The EEG-based methods show various atypical patterns in electroencephalogram of depressed people, such as frontal alpha EEG asymmetry and left frontal hypoactivation (Koo et al., 2017).

Effective treatments for depression, either severe, moderate or mild, are based on psychological therapy and pharmacotherapy. In severe cases of depression, medical practice offers some adjunctive methods of treatment such as the repetitive Transcranial Magnetic Stimulation (rTMS). The left dorsolateral prefrontal cortex (DLPFC), identified as clinically effective in resistant depression treatment, is an area which is mainly targeted in rTMS therapy at a frequency of 10 Hz (Koenigs and Grafman, 2009; Wozniak-Kwaśniewska et al., 2014, 2015). The occurrence of small current in the cortex causes depolarization and hyperpolarization of the neurons triggering neuronal activation. The consequence of this neurostimulation is the modulation of the impaired functional and structural connectivity, associated with depression (mostly brain networks in frontal cortical and subcortical limbic regions; Janicak et al., 2013; Anderson et al., 2016). rTMS is a relatively recent therapeutic approach and has not yet been thoroughly investigated but current evidences show its efficacy in some clinical trials (Markowitz et al., 2010; Janicak et al., 2013; Anderson et al., 2016; Kimiskidis, 2016). In 2008 through efficient meta-analysis, the FDA (Food and Drug Administration) approved rTMS for treatment of patient resistant to antidepressant trials (Markowitz et al., 2010). The data has shown the usefulness of TMS not only for MDD patients but also in treatment of patients with epilepsy, schizophrenia, and other neurological diseases (Kimiskidis, 2016).

It was found that the dynamic organization of brain in depressed patients differ from that in healthy persons (Akar et al., 2015; Anderson et al., 2016), what is manifested in disconnection of links between frontal cortical and subcortical limbic areas. The dysfunctions in these regions, occurring in the microstructure of white matter, are correlated with an inappropriate mood regulation (Anderson et al., 2016). It has been shown that EEG of patients with depression can be distinguished from the EEG of healthy controls using the procedures based on selection of features representative for MDD patients and on their appropriate classification. Different methods have been applied by many researchers, including spectral analysis (such as power spectral density in physiological frequency band; Wozniak-Kwaśniewska et al., 2014, 2015; Liao et al., 2017), Genetic Algorithm (GA), Linear Discriminant Analysis (LDA) (Mohammadi et al., 2015), Discrete Wavelet Transform (Akar et al., 2015), ICA or PCA (Mulders et al., 2015), and also classification methods, based on predictive models, such as the Decision Tree (DT) (Mohammadi et al., 2015), Discriminant Analysis (MacCrimmon et al., 1993), and Artificial Neural Network (Broniec, 2009). Among others, the previous studies have demonstrated higher power spectral density in alpha, theta, and beta bands in some brain areas for MDD patients than for healthy subjects (Olbrich et al., 2014, 2015). However, the study in which the same dataset was used as in this work has shown slightly different results comparing MDD responders and MDD-non-responders to rTMS stimulation (Wozniak-Kwaśniewska et al., 2015). Group of MDD-responders had higher power spectral density (PSD) in delta and theta bands, whereas the lower PSD was found in alpha band as an indicator of improvement of health state (Wozniak-Kwaśniewska et al., 2015). Moreover, PSD in theta and beta bands was higher in prefrontal cortex in BP compared to MDD patients. The impact of stimulation time was investigated also for these data (Wozniak-Kwaśniewska et al., 2015). It was shown that the PSD decreased in left prefrontal areas for delta and theta bands as well as in the contralateral DLFPC for beta and gamma bands. Connectivity methods and indices based on graph theory using EEG data are widely recognized as a valuable research tool. Although, numerous studies have demonstrated utility of EEG connectivity measures (Sporns et al., 2000, 2005; Bassett and Bullmore, 2006, 2009; Stam and Reijneveld, 2007; Stam et al., 2007, 2016; Bullmore and Sporns, 2009; He and Evans, 2010; Stam, 2010; Friston, 2011; Sporns, 2011, 2013), only a few of them concerned the study of brain response to depression treatment with rTMS. To authors' knowledge, only a few study has applied non-linear EEG measures to predict rTMS treatment outcome for depression patients. Arns et al. (2014) found that non-linear measure (Lempel-Ziv Complexity) analyzed in the alpha band allows to distinguish responders and non-responders. Hu (2010) showed that the global phase synchronization index of depression under the states of closed eyes and mental arithmetic is much lower than that of controls.

Another non-linear measure is Higuchi fractal dimension (FD) which was extensively used in the EEG complexity analysis (Klonowski et al., 2000, 2002, 2005, 2006; Olejarczyk, 2007, 2011; Olejarczyk et al., 2009; Zappasodi et al., 2014, 2015; Cottone et al., 2016, 2017). Several authors applied this method to show the differences between EEG complexity of patients with depression and healthy controls (Bahrami et al., 2005; Ahmadlou et al., 2012; Bachmann et al., 2013, 2018; Akdemir Akar et al., 2015). Lebiecka et al. (2018) showed among others that FD decreased after rTMS in MDD-responders, as it was expected. Investigation of the origin of major depression disorder and the influence of rTMS on depression treatment has generated a great number of researches, including studies using EEG-based methods (Rossi et al., 2009; Gaynes et al., 2014; Lefaucheur et al., 2014; Brunoni et al., 2017; McClintock et al., 2018). The main aim of these studies is to understand neural mechanisms of this process and to estimate a treatment progress. In the accomplishment of this goal, the comparison of different connectivity analysis methods is important (Olejarczyk and Jernajczyk, 2017b; Olejarczyk et al., 2017a,c), thus this issue requires further investigation to improve the effectiveness of therapy.

In this paper the impact of rTMS on brain activity in patients with MDD or BP was studied. The therapeutic response was correlated to the evaluation of the changes in brain functional connectivity between consecutive rTMS sessions and also between non-responders and responders to treatment. Comparison between MDD and BP patients was also performed. The functional connectivity was estimated by means of Phase Locking Value (PLV), which is a non-linear measure of the phase relationships between EEG signals recorded at different channels. Thus, the PLV, in contrast to FD, is a measure that provides information about interactions between pairs of EEG channels, and hence allows for investigation of changes in brain connectivity caused by the rTMS. In particular, in this study we tested the hypotheses that: (1) the degree and strength of phase synchronization quantified by PLV change after application of rTMS and these changes are frequency- and topographically-specific as well they depend on time of stimulation; (2) groups of non-responders differ from responders as well as groups of MDD differ from BP. In the first case, a null hypothesis that no changes of data before and after rTMS stimulation was verified for every frequency band, every EEG channel, and every session, respectively. Whereas, in the second case, a null hypothesis that the compared groups are not different was tested.

## Materials and Methods

### Subjects

The EEG data were collected at the Psychiatry Department of Grenoble University Hospital, after approval by the local ethical committee (ID RCB: 2011-A00114-37). All 18 participants gave a written informed consent. The same data were already analyzed using spectral power and complexity analysis (Wozniak-Kwaśniewska et al., 2015; Lebiecka, 2018; Lebiecka et al., 2018).

Ten patients enrolled in the study (6 females, age range 32–69, mean 48.7 ± 12.6) were diagnosed with BP and 8 patients (6 females, age range 44–64, mean 52.1 ± 7.8) suffered from MDD according to Diagnostic and Statistical Manual of Mental Disorder 4th ed (DSM-IV) criteria for Major Depressive Episode (American Psychiatric Association (APA), 2000). Each of these groups were also divided into responders and non-responders to the therapy. Demographic and clinical data for the patient groups are reported in the Tables 1, 2. Exclusion criteria based on detailed interview and medical history were: age under 18 years, drug abuse, previous electroconvulsive therapy, neurological illness, convulsive disorders, current comorbid major mental disorders assessed by clinical examination. The inclusion criterion was no response to pharmacological therapy using minimum two distinctly different classes of antidepressant medications for actual depressive episode occurring at the time of enrolment or earlier.

**Table 1:** Demographic data of the participants: number of patients in every group, average age, and average duration of illness.

		**Number of patients**	**Age**	**Illness duration**
MDD	Response	4	53.3 ± 5.8	10.3 ± 6.1
	Nonresponse	4	51.5 ± 7.5	10.3 ± 5.7
BP	Response	6	49 ± 13	14.8 ± 9.5
	Nonresponse	4	50 ± 10	24 ± 8.7
Total		18	48 ± 9.7	15.1 ± 9.6

**Table 2:** Clinical characteristic of the participants: MADRS response test scores in the successive sessions.

		**MADRS response test**			
		**1st session**	**10th session**	**20th session**	**Δ score**
MDD	Response	33.3 ± 1.8	24.5 ± 6.2	9.3 ± 3.3	−9.0 ± 5.4
	Nonresponse	18.0 ± 5.1	19.0 ± 7.8	–	–
BP	Response	22.0 ± 2.7	13.7 ± 7.2	3.5 ± 3.2	−9.0 ± 4.3
	Nonresponse	25.2 ± 2.2	23.6 ± 4.7	24.0 ± 8.8	−3.6 ± 5.9
Total		24.0 ± 6.3	19.7 ± 7.6	20 ± 10	−4 ± 10

All patients were on a range of medications. For bipolar patients, mood stabilizer medication has been unmodified for at least 2 weeks prior to the entry in the study, and remained unchanged throughout the course of the study. No benzodiazepines were administered two weeks before and during rTMS treatment. For MDD patients, pre-treatment with an antidepressant and/or mood stabilizer medication was kept unmodified for at least 4 weeks prior to the entry in the study, and remained unchanged throughout the course of the study. Only cyanemazine and hydroxyzine were tolerated during the study.

Demographics characteristics (gender and age) and clinical characteristics (illness and episode duration, depression severity) were evaluated for each patient using Montgomery Asberg Depression Rate Scale (MADRS) (Montgomery and Asberg, 1979), 13-item Beck Depression Inventory (BDI-Short Form) (Collet and Cottraux, 1986; Bouvard et al., 1992; Beck et al., 1996), and Clinical Global Impression (CGI). For bipolar patients, maniac or mixed symptoms were evaluated with Young Mania Rating Scale (YMRS) (Young et al., 1978). All patients were assessed at inclusion, before the first EEG recording and after each 5 rTMS sessions by the same senior psychiatrist (David Szekely). The response to rTMS treatment was defined as at least 50% reduction of the baseline MADRS scores. Patients were qualified as remitters when MADRS score was <8. If YMRS was more than 15, at inclusion or during the course of rTMS treatment, patients were excluded from the trial. The absolute changes in MADRS scores between baseline and the end of rTMS (4 weeks after the first evaluation) were used to calculate clinical improvement.

The rTMS sessions were performed according to the standard procedure for depression at Grenoble University Hospital. The left DLPFC was stimulated using a MagPro x 100 TMS stimulator (Tonica Elektronik A/S, Den- mark). Frequency of rTMS was set to 10 Hz and total number of pulses per session amounted to 2,000 continuously at 120% motor threshold. Fourteen patients underwent treatment consisting of 20 sessions within a period of 4 weeks. No patient response to the treatment was observed in eight subjects. It was the cause of reduction of the number of sessions to ten for four of them.

### EEG Registration and Preprocessing

The ~15-min EEG recordings from each of participants were registered before and after 1st, 10th and 20th session, respectively. A 64-channel elastic cap with Ag/AgCl electrode was used (Fast'n'Easy Cap, Brain Products GmbH, Munich, Germany) with a referential montage, where the reference electrode was placed in FCz position. The data acquisition was performed at 2,500 Hz sampling frequency with 16-bit resolution. Participants kept closed eyes and were seated in a reclining armchair with neck supported with a pillow during the EEG signal acquisition.

Preprocessing procedures were performed using EEGlab toolbox in MATLAB environment before the EEG analysis. Firstly, the digitalized data were down-sampled to 250 Hz and band-pass filtered with two-way least-squares FIR filtering. Consecutive two steps were applied: low-pass filtering with cut-off frequency at 45 Hz and high-pass filtering with cut-off frequency at 0.5 Hz. Such prepared data were analyzed visually to mark time segments contained artifacts. Fully automated artifact cancellation methods using Independent Component Analysis (ICA) were also applied for removal of ocular and muscular artifacts.

The best quality 1-min segments of each recording were chosen visually. Data were normalized and segmented into 20–s epochs and they were analyzed in five frequency bands separately: delta (1–4 Hz), theta (3.5–7 Hz), alpha (7.5–13 Hz), beta (14–30 Hz), gamma (30–45 Hz).

### Phase Locking Value

The Phase Locking Value (PLV) is a bivariate method allowing for quantifying interactions between signals. It provides the information about phase coupling between two EEG signals.

The instantaneous phase of signal x(t) can be calculated using the Hilbert transform HT{·} (Rosenblum et al., 1997, 2001; Mormann et al., 2000):

(1) ϕ(t)=arctan(Im{z(t)}Re{z(t)})=arctan(HT{x(t)}x(t));ϕ∈[-π,π]

*z(t)* is analytic signal obtained from *x(t)*:

(2) $$\begin{array}{r} z\left(t\right)=x\left(t\right)+i\cdot HT\left\{x\left(t\right)\right\}=A\left(t\right)\cdot e^{i\cdot \phi \left(t\right)} \end{array}$$

where A(t) and Φ(t) are the amplitude and phase of signal x(t), respectively.

A pair of real signals x_1_(t) and x_2_(t), which phases are Φ_1_(t) and Φ_2_(t) respectively, are synchronized if their relative phase Φ_12_(t) is constant:

(3) $$\begin{array}{r} \phi_{12}\left(t\right)=\phi_{1}\left(t\right)-\phi_{2}\left(t\right)=const. \end{array}$$

The PLV can be computed as Aydore et al. (2013) and Niso et al. (2013):

(4) $$\begin{array}{l} PLV=\left|\frac{1}{N}\sum_{j=0}^{N-1}e^{i\phi_{12}(j\Delta t)}\right|={(\left[\frac{1}{N}\sum_{j=0}^{N-1}\sin{(}^{\;}\phi_{12}(j\Delta t))\right]}^{2}\\ \;+{{\left[\frac{1}{N}\sum_{j=0}^{N-1}\cos{(}^{\;}\phi_{12}(j\Delta t))\right]}^{2})}^{1/2} \end{array}$$

where *i* is the imaginary unit, Δt is the time interval between two successive samples and N is the total number of samples.

The PLV ranges from 0 to 1. The lower PLV, the less likely there is any phase synchronization between two signals. The value 1 describes situation when relative phase is constant.

The PLV calculations were performed using HERMES toolbox (Niso et al., 2013), which provides numerous commonly used linear and non-linear indexes of functional and effective connectivity with dedicated visualization methods.

### Indices Based on Graph Theory

In graph theory the brain is modeled as a graph composed of nodes, representing brain regions or simply by EEG channels, and links between them, representing functional connections determined here by PLV. For each of the graphs, the basic indices, such as degree and strength, were calculated (Rubinow and Sporns, 2010) to detect connectivity patterns characteristic for different brain states before and after TMS depending on kind of disorder and on patient responsiveness as well as on time of stimulation. These indices are the starting point for determining other more complex indices such as indices of integration, separation, centrality, resilience (Rubinow and Sporns, 2010) as well as inter-hemispheric, and fronto-posterior asymmetry (Olejarczyk and Jernajczyk, 2017b).

The *degree* of an individual node is equal to the number of links connected to that node. Thus, this index reflects the importance of a node in the network. The degree is one of the most used measures of centrality concerning importance of individual nodes in interaction with other nodes in the network. The high-degree nodes (hubs) play a key role in network resilience and facilitates functional integration, i.e., they have an ability to rapidly combine specialized information from different brain regions (Rubinow and Sporns, 2010). The weighted variant of the degree, termed the *strength*, is defined as the sum of all neighboring link weights (Van Wijk et al., 2010; Bassett and Lynall, 2013) connected to the node. The strength of k-th node is defined as:

(5) $$\begin{array}{r} strength_{k}=\;\sum_{i}^{K-1}c_{ik} \end{array}$$

where *K* is a total number of nodes and *c*_*ik*_ is a weight of link between node *k* and *i*.

To perform the calculations of these indices, every graph was presented in the form of a matrix of connections between every pair of EEG channels, so called adjacency matrix. All adjacency matrices constructed from PLVs computed from each recording were thresholded and tested against a null model separately. To evaluate the statistical significance of these PLVs matrices the standard procedure was performed using HERMES software (Niso et al., 2013). In this case, the results of PLV calculated for 63 EEG electrodes were stored for each subject in a 63 × 63 matrix. When the multivariate surrogate data test was performed an additional 63 × 63 matrix of *p*-values was produced for each subject. The null hypothesis of independence of the time series was tested at the statistical significance level *p* = 0.05. All non-statistically significant values of PLV matrix were set to zero.

The results of all-to-all functional connectivity were visualized using the MNE toolbox, an open-source Python software for analyzing and visualizing neurophysiological data (Gramfort et al., 2013, 2014). Connectivity circle plot bases on PLVs connectivity matrices between all pairs of 63 EEG electrodes. Such graphical presentation of functional EEG connectivity was applied for the first time in this paper. The changes between both brain states, before and after rTMS stimulation, were visualized in form of differential plots, i.e., in place of the PLV values in each of both states, the differences between them were shown.

### Statistical Analysis

The following procedures were performed to evaluate the effect of rTMS in the four groups of patients as well as to find differences between them.

The size of the datasets was not equal in all sessions. In the last, third session the data were acquired only in 13 from 18 patients. Thus, the PLV was calculated for each recording from 2 sessions in group of 18 patients and from 3 sessions in group of 13 patients. The significance of PLV values from each matrix was determined using surrogate data analysis (Theiler et al., 1992). The amplitudes of signals were maintained while their frequency relationships were modified by shuffling of the data in frequency domain. The signal values were then obtained again from surrogate data transformed to time-domain. The number of surrogates was set to 100 which is a sufficient value for chosen *p*-value. The adjacency matrices were constructed from data with *p*-value below the 0.05 threshold. Then, these matrices were analyzed using indices based on graph theory. For each of the graphs, the indices degree and strength were calculated.

Multivariate analysis of variance (ANOVA) is a statistical test used to assess the impact of many independent variables (factors) on the value of the dependent variable. The ANOVA can be performed if the following two assumptions are fulfilled: (1) each population has a normal distribution, and (2) variances in populations are equal. To verify these assumptions the Shapiro-Wilk test was carried out before the analysis of variance was performed.

The five following factors were considered: GROUP (MDD-responders, MDD-non-responders, BP-responders, BP-non-responders), BAND (delta, theta, alpha, beta, gamma), CONDITION (baseline or after sessions), CHANNEL (63 channels), SESSION (1st, 10nd, and 20th session) for applying the ANOVA analysis in several steps.

**Figure 1:**
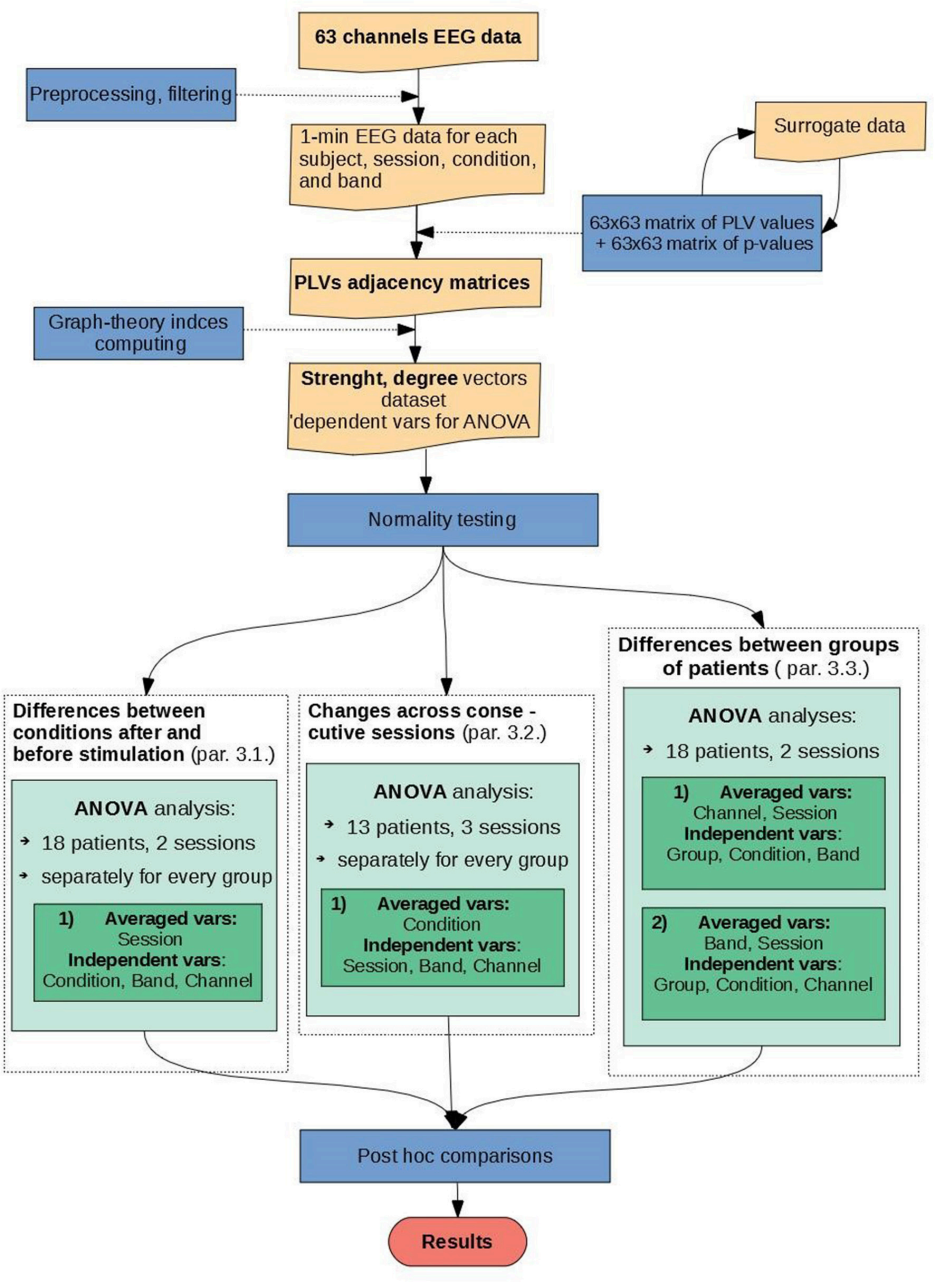
Block diagram illustrating the individual steps of EEG signal analysis.

Firstly, statistical analysis was carried out for data of 18 patients and 2 sessions conducting ANOVA tests with factors: BAND, CONDITION, and CHANNEL to investigate the impact of rTMS stimulation on functional connectivity. The adjacency matrices were analyzed separately by every group and averaged over all sessions (see Figure 1, left green block). Secondly, the impact of time stimulation was analyzed using data from 13 patients in 3 sessions. For this purpose, the ANOVA with factors: SESSION, BAND, and CHANNEL in each condition and in each group separately was performed (see Figure 1, middle green block). Finally, to investigate differences between groups, three factors were used: BAND, CHANNEL, and CONDITION separately for every pair of groups. Four pairs were compared: MDD_responders vs. MDD_non-responders, BP_responders vs. BP_non-responders, MDD_responders vs. BP_responders and MDD_non-responders vs. BP_non-responders (see Figure 1, right green block).

Analysis of variance is a test that allows to verify the zero hypothesis H_0_ of the equality of mean values in the considered populations having a normal distribution with equal variances at fixed level of significance α. If we reject the null hypothesis, we accept an alternative H_1_ hypothesis that at least two averages are not equal. In the analysis of variance, the most commonly used distribution is the right-skewed asymmetric F distribution, which is the ratio of two chi-square distributions. The shape of each chi-square distribution depends on degrees of freedom. The verification of hypotheses should be carried out in such a way that ensure the lowest probability of making a mistake. Two types of errors should be considered to decide whether reject the null hypothesis, i.e., type I error consisting in rejection of a true null hypothesis with the probability equal to the significance level α, and type II error consisting in accepting a false null hypothesis. In order to verify the type II error, the calculated value of the *F*-test should be compared with the statistical limit value read out from the tables for a given significance level α and for a given number of degrees of freedom of each factor of one or two-way ANOVA. If the calculated *F* is greater than the limit of the F statistic, then the null hypothesis H_0_ should be rejected in favor of the alternative H_1_ hypothesis (otherwise there is no reason to reject H_0_).In this study, the type II error was verified using the probability distribution calculator provided by the STATISTICA software.

## Results

### Differences Between Conditions

**Figure 2:**
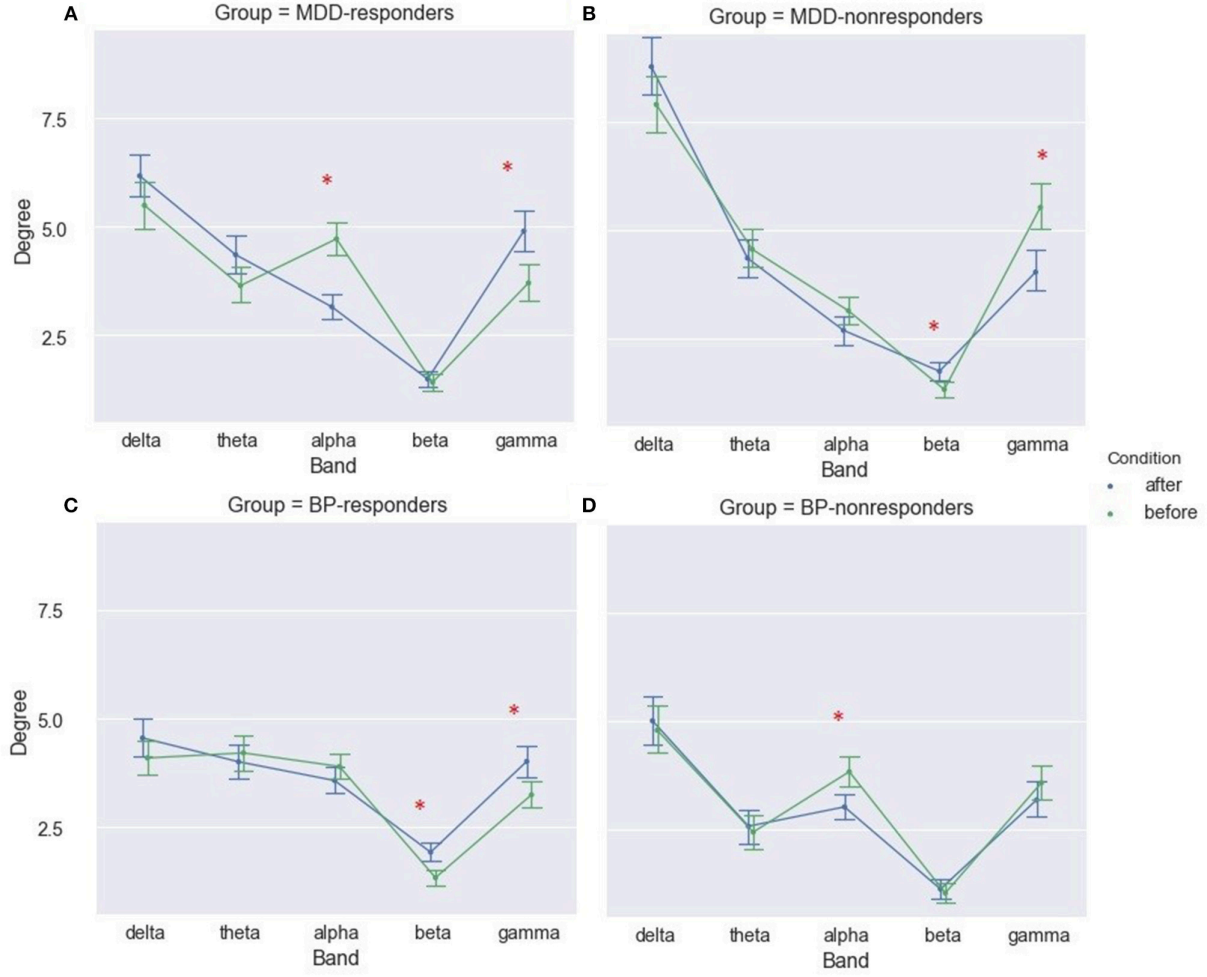
Degree of PLV for MDD-responders **(A)**, MDD-nonresponders **(B)**, BP-responders **(C)**, and BP-nonresponders **(D)** for each EEG band before and after rTMS stimulation. The significant differences are marked by asterisks.

No significant differences in strength and degree of PLV before and after stimulation were found for any of the four groups. However, when the indices after stimulation were compared to the baseline for the five frequency bands separately, significant differences were found for some of them in all groups. In the BP-responders group the increase of two indices (strength and degree) occurs in beta and gamma bands after stimulation (degree index only in Figure 2C). MDD-responders group had overall higher strength and degree indices in gamma after stimulation (Figure 2A for degree index). The index strength increased also in delta band after stimulation. Moreover, the decrease of both indices in the alpha band was observed. The ANOVA results for MDD-responders and BP-responders are summarized in Tables S1–S4.

The results for both groups of non-responders were different than the results for the responders. A significant decrease of degree and strength after stimulation in gamma band and increase of these indices in beta band have been observed for MDD-non-responders (Figure 2B for degree index). Moreover, the index strength was lower after stimulation in alpha band for BP- non-responders (not shown).

The biggest differences in strength of PLV between conditions (before and after rTMS stimulation) were in gamma bands for MDD-responders (Figure 2A for degree index) and in beta and gamma bands for BP-responders (Figure 2C for degree index) since they were illustrated in form of graphs. The all-to-all functional connectivity for significant differences between conditions have been visualized as circular graphs. Such visualization provided information about interdependencies and their strength between EEG channels. Only the 30 strongest PLV connections were considered to show the differences between conditions.

**Figure 3:**
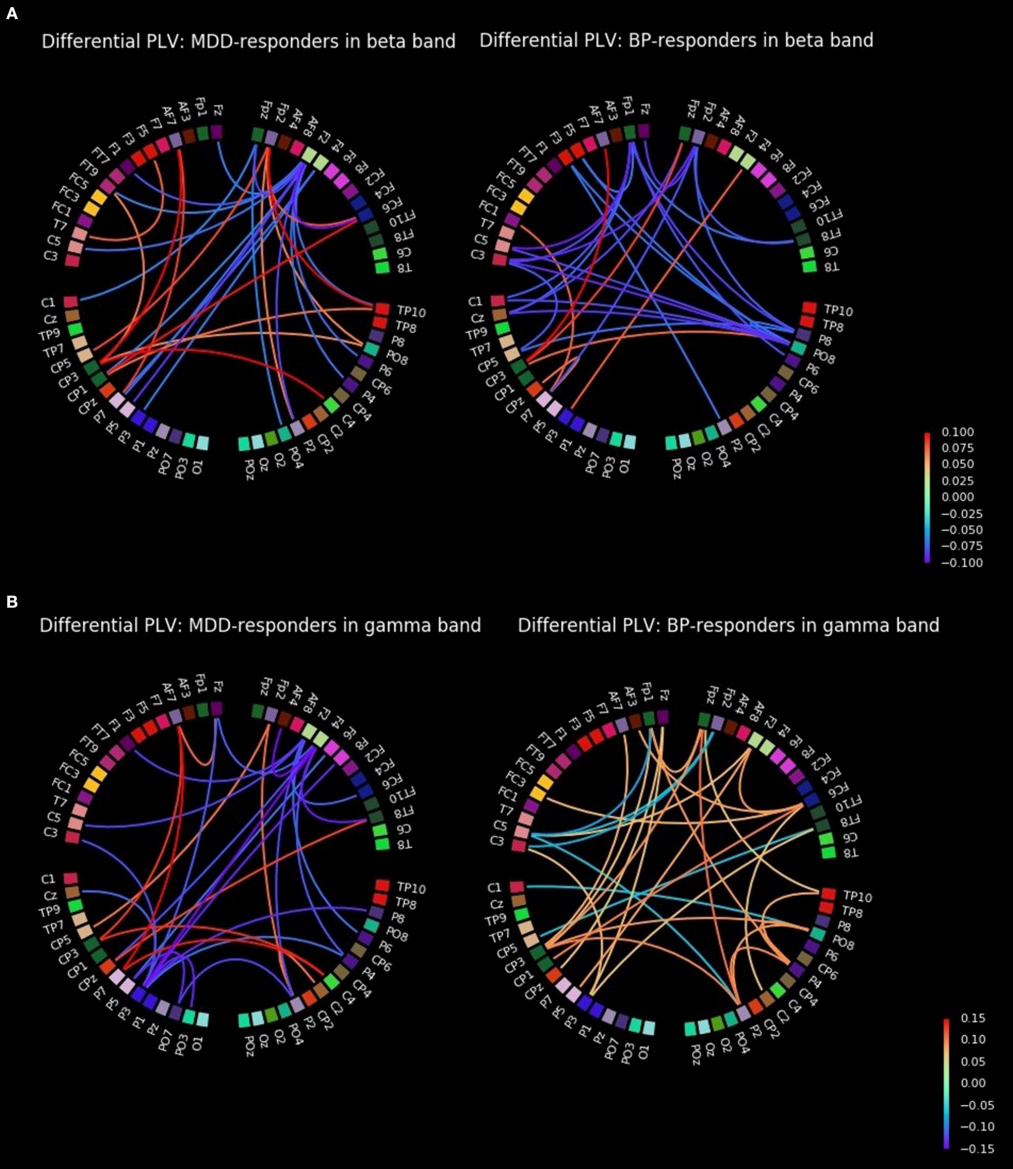
All-to-all differential connectivity of PLV (30 strongest connections before and after rTMS stimulation) for responders groups in beta **(A)** and gamma bands **(B)**.

The graphs for beta band are presented in Figure 3A. They provided information about increase of strength between some of right centro-parietal (CP3, CPz) and parietal areas (P3, P7) and mostly frontal channels (AF7, FC1, Fpz, F4) in BP-responders. Whereas, in MDD-responders except the strengthening the connections between centro-parietal (CP1, CP3, CP5) or parietal (P3, P5, P7) areas and the frontal ones (Fp2, AF7, FT9, FC6), more additional connections appeared with right hemisphere (C4, PO8, TP10, FC6). Moreover, in MDD-responders the frontal and left temporal areas were more interconnected (F5-T7, Fp2-FC6). Some hubs are clearly visible in both graphs as well. In MDD-responders the strength of hub placed at F2 decreased after TMS, while in BP-responders a decrease of strength was seen mainly in right parietal (P8, PO8), left central (C5, C3, C1, Cz, CP5), and frontal (Fp2, Fp1) areas.

The graphs for gamma band are presented in Figure 3B. The strength of PLV between right central (C2, C4), left centro-parietal (CP5, CP3, CPz, P7), and frontal (AF7, Fp2, FT8) areas increased for MDD-responders. A decrease of strength was observed in some hubs in frontal (Fz, F2, F4) and left parietal (P3, P5) areas as well. BP-responders showed a general increase of connectivity in various areas with hubs in centro-parietal (CP3, CP1, Cpz, P5, P3) and frontal (AF7, AF3, Fp1, Fz, Fpz, F2, FC6) and also right parietal areas (P2, P4, PO8, TP10) areas in gamma band.

### Changes Across Consecutive Sessions

To investigate the interdependencies between sessions the three-way ANOVA with factors: SESSION, BAND, and CHANNEL was applied.

The dataset recorded in 13 patients before rTMS stimulation and after 1st, 10th, and 20th session was analyzed to evaluate the influence of stimulation time.

**Figure 4:**
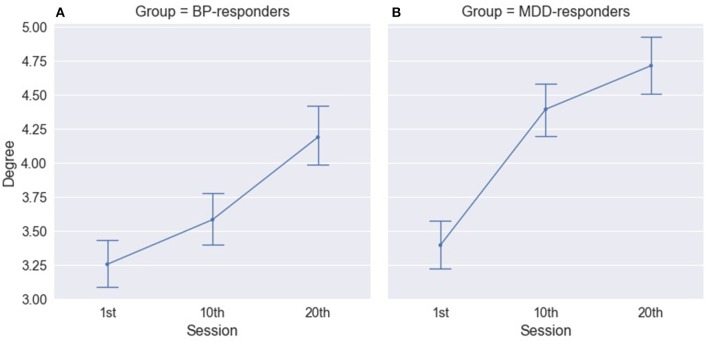
Degree of PLV for BP-responders **(A)** and MDD-responders **(B)** in consecutive sessions.

The three-way ANOVA results revealed significant differences for factor SESSION in both groups of responders: MDD- responders and BP-responders. The significant increase in degree index have been observed between all sessions for BP-responders (Figure 4A, Table S5) and between 1st and 10th and between 1th and 20th sessions for MDD-responders (Figure 4B, Table S5). A similar difference was found for strength index except differences between 1st and 10th session for BP-responders (Table S6). No significant differences for MDD-non-responders and BP-non-responders have been observed.

The differences between consecutive sessions in each of group were also analyzed for different EEG frequency bands. The results obtained for both degree and strength indices were very similar. Therefore, the results of strength index analysis only are provided.

**Figure 5:**
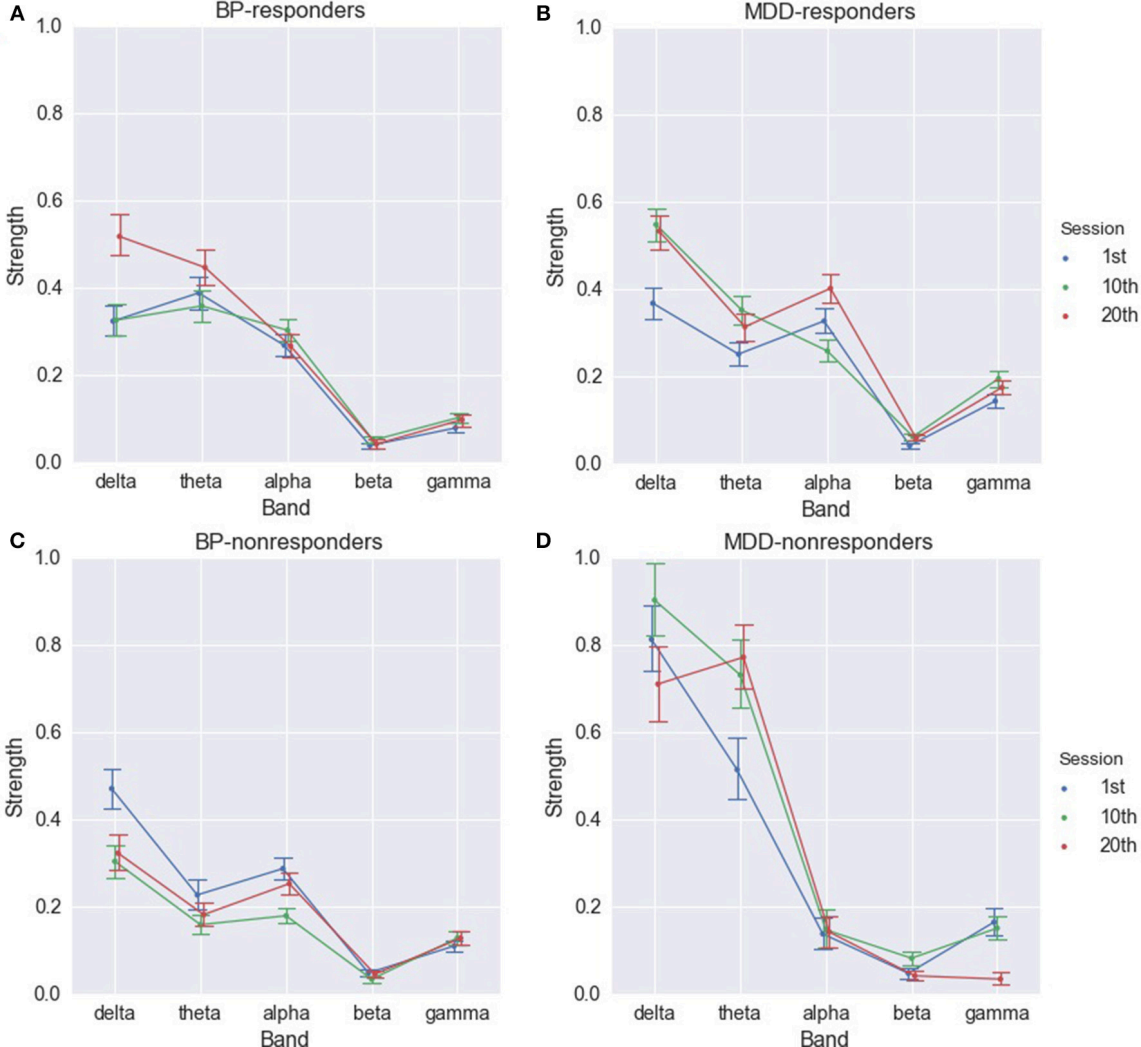
Strength of PLV for BP-responders **(A)**, MDD-responders **(B)**, BP-non-responders **(C)**, and MDD-non-responders **(D)** in consecutive sessions in each of frequency bands.

For MDD-responders a significant increase was observed between 1st and 10th session in all bands, except alpha band (Figure 5B and Table S7). The strength of alpha band decreased between 1st and 10th session, but the changes were not permanent. Then, a significant increase of strength index in alpha band occurred between 10th and 20th session.

The increase in delta, theta, and gamma rhythms in function of the number of sessions was observed also for BP-responders. The indices in delta and theta rhythm are higher between 1st and 20th as well as 10th and 20th session (Figure 5A and Table S8). The significant differences between 1st and 10th, 1st and 20th session occur in gamma band. In alpha band indices were significantly higher in the 10th session.

The results for non-responders were different. In MDD-non-responders group a significant increase between 1st and 10th, 1st and 20th session only in theta and beta bands occurred (Figure 5D and Table S9). However, between 10th and 20th session the index dropped for beta band to the previous value. The index decreased also in delta and gamma bands between 10th and 20th session (Table S9).

In BP-non-responders the degree and strength indices decreased in delta, theta, alpha, and gamma bands mainly between 1st and 10th session. Comparing the differences between 1st and 10th with differences between 10th and 20th sessions revealed that the longer stimulation caused an opposite effect to the shorter stimulation (Figure 5C and Table S10).

**Figure 6:**
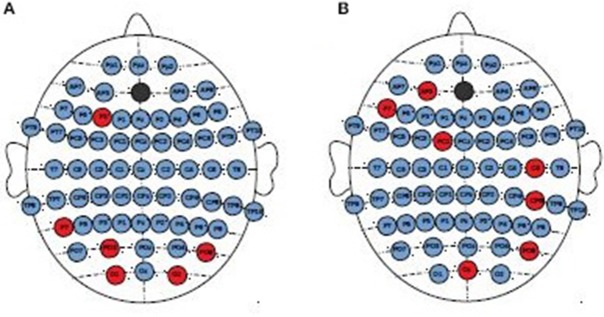
Topographical differences between degree of PLV in consecutive sessions for MDD-responders **(A)** and BP-responders **(B)** in delta band. An increase of degree was marked with red color.

No significant differences between consecutive sessions were observed in the individual EEG channels when whole frequency range was considered. Whereas, the topographical analysis between consecutive sessions for different frequency bands showed increase of strength index in delta band for MDD-responders (Figure 6A) and BP-responders (Figure 6B). The significant and permanent increase of degree index (between 1st and 10th or 1st and 20th or 10th and 20th session) was marked with red color. No significant results have been seen for other bands.

### Differences Between Groups of Patients

Finally, to find the differences between groups of patients, the three-way ANOVA with factors: GROUP, CONDITION, and BAND was performed. The following groups of patients were compared: MDD-responders vs. MDD-non-responders; BP-responders vs. BP-non-responders; MDD-responders vs. BP-responders, and MDD-non-responders vs. BP-non-responders.

#### MDD-Responders vs. MDD-Non-responders

**Figure 7:**
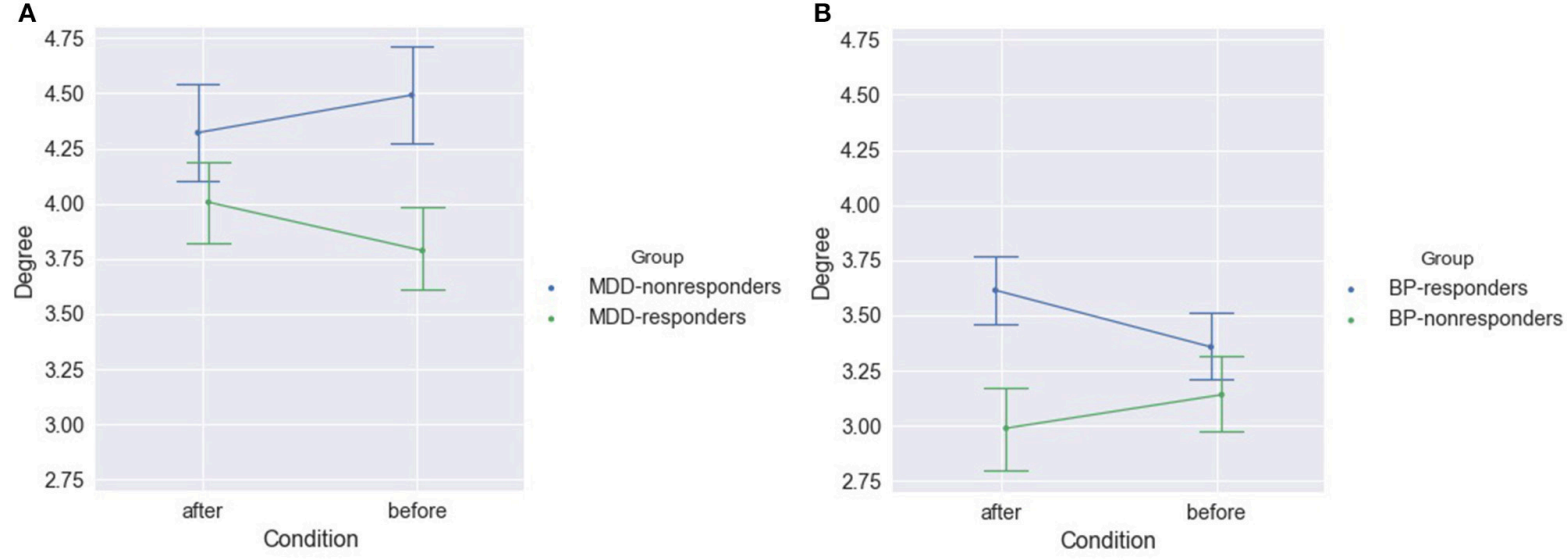
Degree of PLV for MDD-responders and MDD-non-responders **(A)** and for BP-responders and BP-non-responders **(B)** after and before stimulation.

In MDD-responders patients the indices degree and strength were lower for responders than for non-responders before as well as after stimulation (c.f. Figure 7A and Table S11 for degree index). However, MDD reacted in a different way than BP to the rTMS stimulation. The difference between responders and non-responders was bigger after stimulation than before stimulation for BP group. An opposite effect was observed in MDD group. The degree of PLV was significantly higher in MDD patients than in BP patients independently on the condition (compare Figures 7A,B).

**Figure 8:**
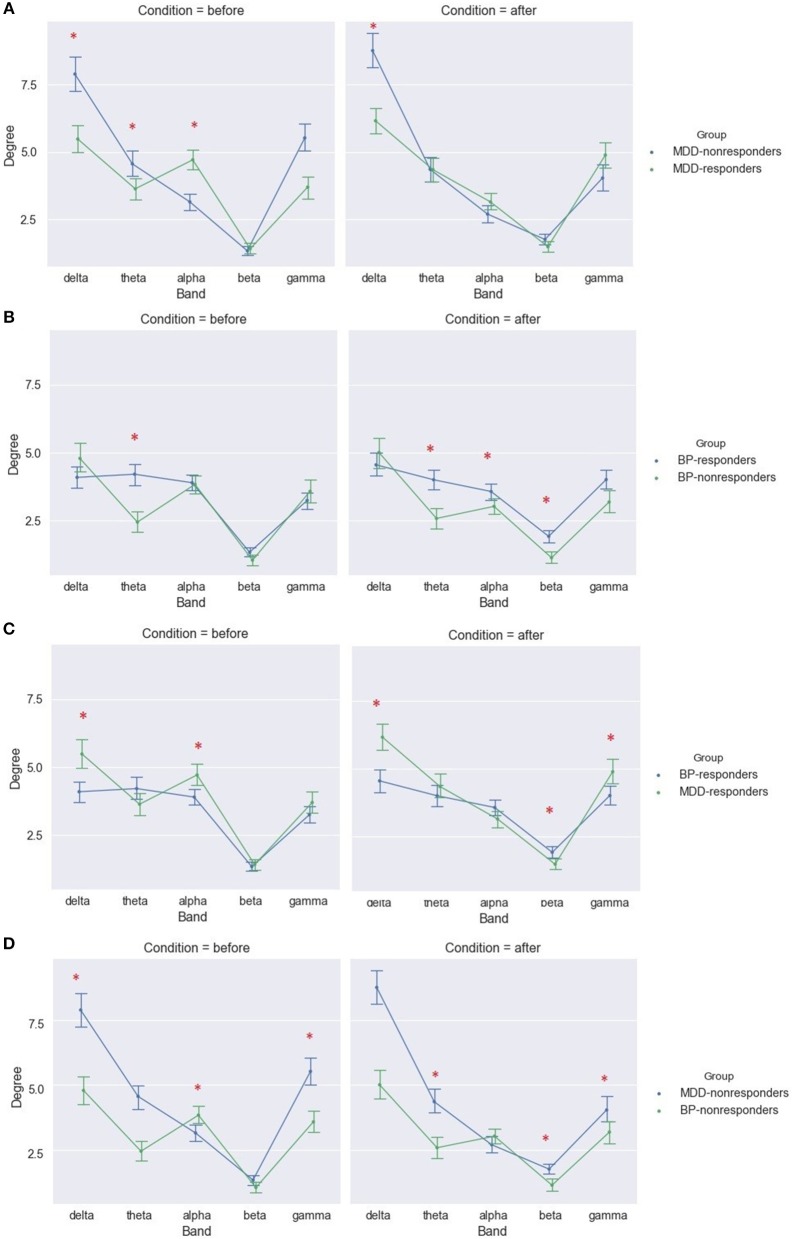
Degree of PLV for MDD-responders and MDD-non-responders **(A)**, BP-responders and BP-non-responders **(B)**, MDD-responders and BP-responders **(C)**, MDD-non-responders, and BP-non-responders **(D)** before and after stimulation in each of frequency bands. The significant differences between groups are marked by asterisks.

The analysis considering each of frequency band separately showed significant differences between MDD-responders and MDD-non-responders before as well after stimulation. The degree index was higher for MDD-responders in alpha band before stimulation. The lower degree index for MDD-responders compared to MDD-non-responders was in delta band before and after stimulation as well as in theta bands before stimulation (Figure 8A). The results for strength index were similar, except for alpha band, for which no significant differences were detected between groups after stimulation (Table S12).

**Figure 9:**
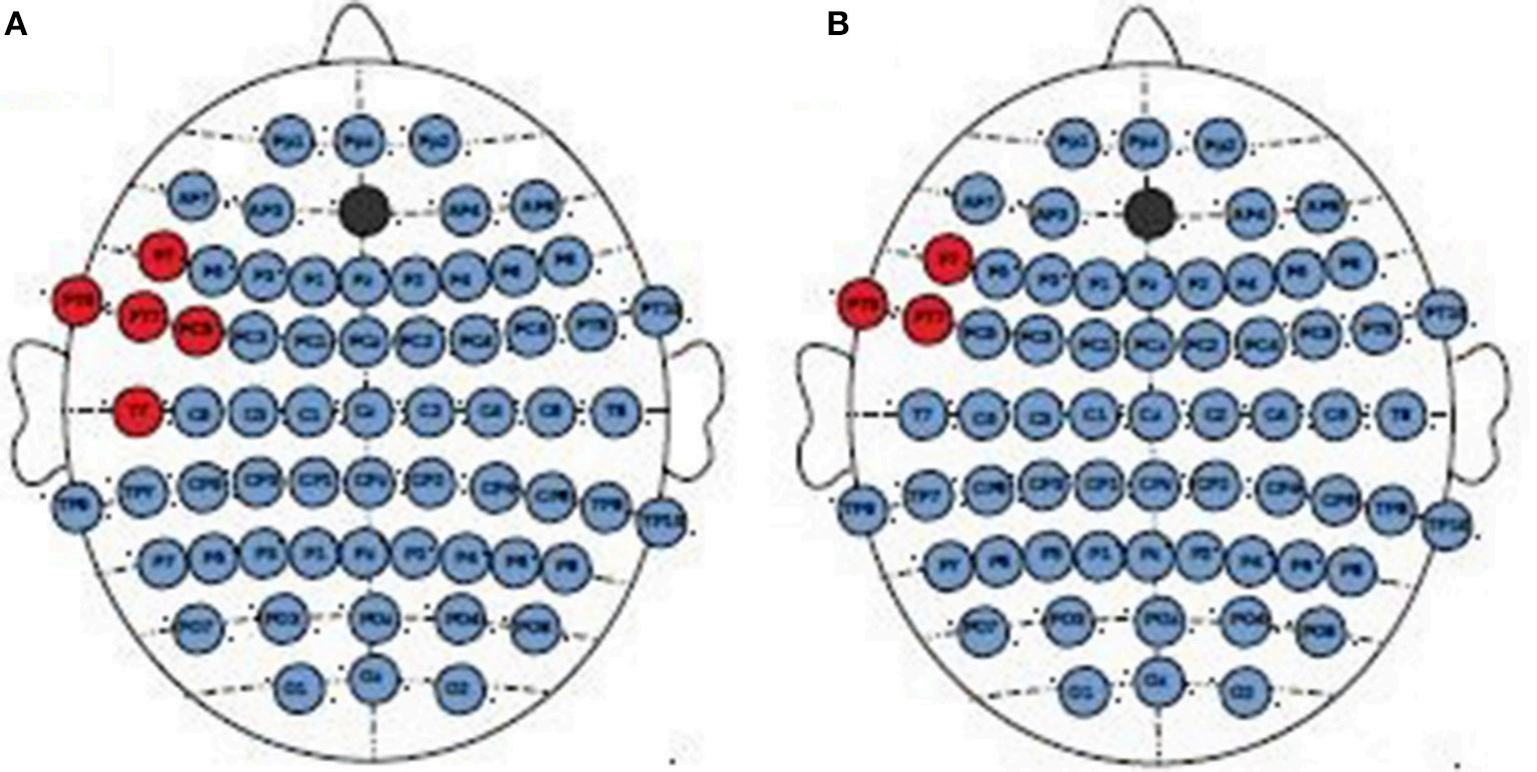
Topographical differences between MDD-non-responders and MDD-responders after stimulation. The higher degree **(A)** and strength **(B)** indices in MDD-non-responders than in MDD-responders were marked in red color.

The topographical differences were found between MDD-responders and MDD-non-responders for indices degree (Figure 9A) and strength (Figure 9B). After stimulation the degree and strength indices had lower values in MDD-responders than in MDD-non-responders at several channels in left frontal area (channels marked in red color on Figure 8).

#### BP-Responders vs. BP-Non-responders

After stimulation the degree index was significantly higher for responders than non-responders in group of BP patients (Figure 7 and Table S13) when all frequency bands were considered.

The analysis of differences between BP groups for different frequency bands showed higher values in theta in both conditions and in beta after stimulation in BP-responders (Figure 8B and Table S14). The similar results were obtained for degree index.

No topographical differences were found between both groups of BP patients.

#### MDD-Responders vs. BP-Responders and MDD-Non-responders vs. BP-Non-responders

Differences between subjects suffered from MDD and BP disease were also investigated separately for responders and non-responders. The significant differences between these groups were found in each of frequency bands in both conditions.

The responders showed significant higher strength index for MDD than for BP group in delta and gamma band in both conditions as well as in alpha band before stimulation. Moreover, the strength index was lower in MDD-responders in beta band after stimulation (Figure 8C). The similar results were obtained for degree index (Tables S15, S16).

The differences between both diseases were found also in the group of non-responders. The degree index was higher for MDD-non-responders in theta and gamma before as well as after stimulation. Moreover, before stimulation the degree index was lower for alpha band in MDD-non-responders than in BP-non-responders (Figure 8D and Table S17). The results for strength index were similar.

**Figure 10:**
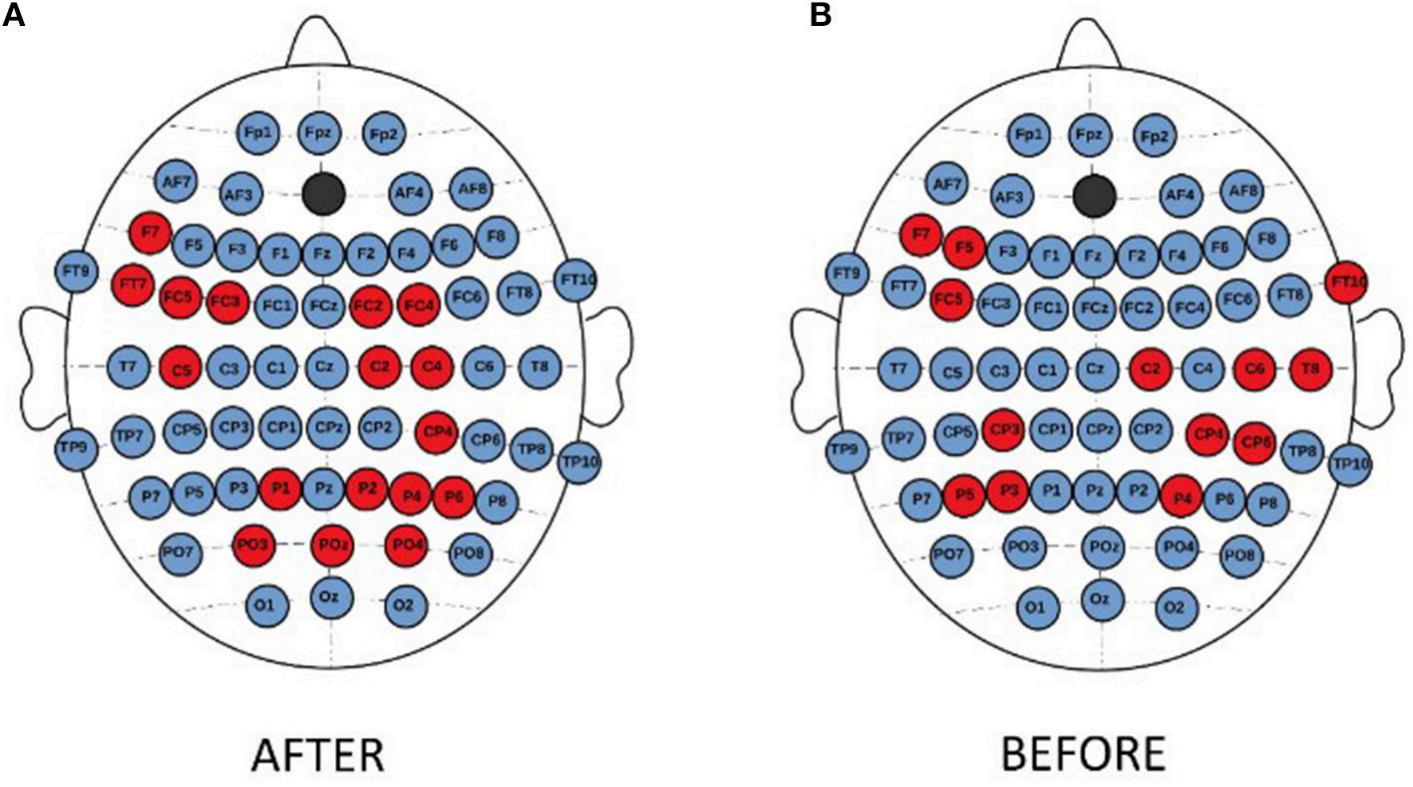
Topographical differences in degree of PLV between MDD-non-responders and BP-non-responders after **(A)** and before **(B)** stimulation. The higher degree in MDD non-responders than in BP-non-responders was marked with red color.

The topographical differences were found between both groups of non-responders. The degree and strength indices were higher for MDD-non-responders than for BP-non-responders in left frontal (F7, FC5) and right centro-parietal areas (C2, CP4, P4) independently on condition. The topographical differences for degree index are shown in the Figure 10.

## Discussion

Despite many studies carried out so far, the understanding of pathophysiology of depression still needs systematic researches for providing a consistent neuropsychophysiologic model of this disease. The main goal of this work was to investigate and elucidate the organization of neural networks implicated in the pathogenesis of major depressive disorder and bipolar depression. In this study, the graph-theory based connectivity analysis was performed using PLV, a non-linear measure quantifying phase interdependencies between the EEG signals, recorded in MDD and BP patients subjected to repetitive transcranial stimulation. The rTMS stimulation was targeted to the left DLPFC, which is functionally connected to the limbic areas of the brain that is highly involved in regulation of mood (Kito et al., 2016). The study of the dynamical properties of brain activity could permit for better prediction of treatment outcome and help to discriminate patients from healthy subjects. The effect of the rTMS therapy was evaluated by the comparison of the analyzed measures (degree and strength of PLV) before and after stimulation.

Frequency-dependent differences in the reactivity to the rTMS therapy were found between four groups of patients. The analysis of PLV indices in each frequency band separately showed their increase in both groups of responders after stimulation in gamma band. The indices increased also in beta band for group of BP-responders. This finding is similar to results obtained by Olbrich et al. (2014, 2015) who analyzed the Phase Lag Index (PLI), another measure of synchronization. They reported that the PLI increased after rTMS for beta band in some prefrontal areas. Other authors found an increase of gamma band power in left DLPFC, which correlated with the improvement in depression symptoms in MDD patients subjected to rTMS stimulation (Kito et al., 2014; Pathak et al., 2016). The results for gamma bands are in line with results of Bailey et al. (2018) who found an increase of connectivity evaluated in gamma band using weighted PLI in MDD-responders 1 week after rTMS therapy. We observed a decrease of PLV indices (degree and strength) in alpha band for MDD-responders also. The results obtained in other studies were ambiguous. Ulrich et al. (1984) observed the reduction of alpha activity, whereas other studies demonstrated increase of global alpha activity in MDD patients (Knott et al., 2001; Leuchter et al., 2012; Olbrich et al., 2014, 2015; Fingelkurts and Fingelkurts, 2015).

The analysis of topographical differences between conditions showed an increased connectivity after stimulation in left/right frontal, left centro-parietal/parietal, and right central/parietal channels in gamma band for MDD responders. In BP-responders an increased connectivity was observed mainly between frontal and left centro-parietal or parietal areas in beta and gamma band. Moreover, the right hemisphere reacted strongly to rTMS in gamma band.

Additional information has been found investigating the alteration of the PLV indices across the consecutive sessions. The indices increased in delta and theta bands for both groups of responders. The changes in delta band was manifested in topographical increase of PLV in left frontal, parieto-occipital/occipital areas in delta band for both groups of responders, and also in right central area only for BP-responders. These results are partly in line with the study of Park et al. (2007). The authors performed the connectivity analysis using another non-linear measure (Synchronization Likelihood). They found a greater synchronization in delta band in healthy persons than in MDD patients, particularly in frontal and left temporal areas, which means that the impact of rTMS manifesting in the increase of indices in the delta band was beneficial.

The analysis of PLV indices allowed to find the differences between groups of patients. The PLV indices were greater in gamma band after rTMS for both groups of responders in relation to non-responders. Whereas, for delta band the indices were greater in responders than in non-responders before as well as after stimulation.

Comparing MDD and BP patients revealed higher phase synchronization of delta and gamma bands in MDD than in BP independently on the responsiveness and on the condition. Whereas, the indices for alpha band before stimulation were lower in MDD-non-responders than in BP-non-responders but higher in MDD-responders than in BP-responders. Moreover, the indices were bigger in theta band in both conditions in MDD-non-responders than in BP-non-responders but lower for MDD-responders than for BP-responders in beta band after stimulation.

This study reuses the data presented also in other papers where spectral power (Wozniak-Kwaśniewska et al., 2015) and Higuchi's Fractal Dimension (Lebiecka et al., 2018) were used. Every of these measures carries other information about the signal. Spectral power density (PSD), in contrast to PLV, gives an information on amplitude of the EEG signal but does not consider nether its phase nor phase dependencies between signals from different channels. Whereas, FD, another non-linear method, which was used in study analyzing the same dataset (Lebiecka, 2018; Lebiecka et al., 2018), is a measure of signal complexity. Both, PSD and FD, do not give information about interactions between signals from different channels. Thus, all these methods should be considered as independent, complementary markers of brain state changes under influence of rTMS. Nevertheless, the comparison of different measures allows to find relationships existing between them. For example, it was found that an increase of phase synchronization (PLV) after rTMS in higher frequencies (beta and gamma bands) in MDD-responders corresponded to a decrease of the EEG signal complexity (FD) in this group of patients (Lebiecka et al., 2018).

## Conclusions

In this paper, the impact of rTMS on the EEG connectivity evaluated by PLV was studied for the first time. The main findings were that the PLV indices were increased in function of stimulation time for both disease groups. Thus, the PLV might be a good marker of recovery from depression.

Our results suggest that the rTMS of left DLPFC caused the increase of the activity in the left DLPFC and other regions functionally connected to this area of the brain. The topographical analysis showed an increase of connectivity between left frontal and right parieto-occipital areas of brain after stimulation. Therefore, this increase of phase synchronization might be considered as a indicator of response to depression treatment.

These results will need to be replicated on a larger and better standardized cohort because one of the limitations of this study is a small number of subjects in all groups. Moreover, the patients under examination were not drug free, which could have an influence on the brain activity. The k-NN algorithm (Olejarczyk et al., 2012; Jozwik, 2013; Olejarczyk, 2018) could be applied to evaluate the error of subjects' classification to one of four groups of patients, basing on the PLV indices. Such approach would allow also to choose a set of the best features (in our case the set of PLV indices). In this study the principal indices (degree and strength) were considered only. Future research will include other indices like measures of integration (clustering coefficient, local efficiency, modularity), separation (characteristic path length, global efficiency), betweenness centrality, resilience (Rubinow and Sporns, 2010) as well as inter-hemispheric, and fronto-posterior asymmetry (Olejarczyk and Jernajczyk, 2017b). Such plenty of indices and factors considered in this study justifies even more the application of the k-NN algorithm. Furthermore, the dependence of other indices on degree should be considered. The indices should be analyzed in function of threshold (Olejarczyk and Jernajczyk, 2017b; Olejarczyk et al., 2017a). The results of classification errors for different thresholds using k-NN method could be compared also. Finally, the directional measures of brain connectivity (Olejarczyk and Jernajczyk, 2017b; Olejarczyk et al., 2017a,c; Zuchowicz, 2018) should be considered in the future studies to better understand the mechanism of action in depression.

## Data availability

The datasets for this manuscript are not publicly available because of Grenoble University Hospital policy. Requests to access the datasets should be directed to Prof. Olivier David, email: Olivier.David@inserm.fr.

## Ethics Statement

This study was carried out in accordance with the recommendations of Ethics Committee of the Grenoble University Hospital with written informed consent from all subjects.

## Author Contributions

UZ: EEG data preprocessing and analysis and writing the manuscript; AW-K: EEG data acquisition; DS: patients recruitment and design of the trial; EO: conception of the work, supervision of the EEG data analysis, and wrote the manuscript; OD: design of the trial, interpretation of EEG data, and critical revision of the manuscript.

### Conflict of Interest Statement

The authors declare that the research was conducted in the absence of any commercial or financial relationships that could be construed as a potential conflict of interest.
