# Supplementary material for: EEG Phase Synchronization in Persons With Depression Subjected to Transcranial Magnetic Stimulation
Source: Front Neurosci. 2019 Jan 14;12:1037. doi: 10.3389/fnins.2018.01037 (PMC6340356; doi:10.3389/fnins.2018.01037)
Supplement: Supplementary file 1 [file Data_Sheet_1.pdf]

Supplemental material

Table S1. One-way ANOVA analysis results for degree index before and after stimulation for five frequency bands in MDD-responders. The critical value of F is equal to 7.7086.

Band	ANOVA results		mean ± std	
	F-value	p-value	before	after
delta	3.543	0.060	5.48 ± 0.26	6.16 ± 0.24
theta	5.603	0.018	3.63 ± 0.20	4.34 ± 0.22
alpha	40.414	0.000	4.71 ± 0.18	3.15 ± 0.15
beta	0.309	0.578	1.412 ± 0.096	1.488 ± 0.096
gamma	13.591	0.000	3.69 ± 0.21	4.89 ± 0.24

Table S2. One-way ANOVA results of degree index of PLV analysis before and after stimulation in five frequency bands in BP-responders. The critical value of F is equal to 7.7086.

Band	ANOVA results		mean ± std	
	F-value	p-value	before	after
delta	2.404	0.121	4.09 ± 0.19	4.55 ± 0.22
theta	0.531	0.466	4.20 ± 0.20	4.55 ± 0.22
alpha	2.330	0.000	3.89 ± 0.14	3.57 ± 0.15
beta	17.145	0.000	1.34 ± 0.083	1.92 ± 1.11
gamma	10.664	0.001	3.24 ± 0.15	4.01 ± 0.17

Table S3. One-way ANOVA results for strength index analysis before and after stimulation for each of five frequency bands in MDD-responders. The critical value of F is equal to 7.7086.

Table 5.9. One-way ANOVA results for strength index analysis before and after stimulation for each of five frequency bands in MDD-responders.

Band	ANOVA results		mean ± std	
	F-value	p-value	before	after
delta	4.434	0.035	0.43 ± 0.20	0.48 ± 0.018
theta	4.939	0.026	0.276 ± 0.015	0.326 ± 0.016
alpha	40.581	0.000	0.0352 ± 0.014	0.231 ± 0.012
beta	0.089	0.765	0.0518 ± 0.0035	0.0503 ± 0.0032
gamma	11.401	0.001	0.1474 ± 0.0085	0.1897 ± 0.0092

Table S4. One-way ANOVA results of strength index analysis before and after stimulation for each of five frequency band in BP-responders. The critical value of F is equal to 7.7086.

Table 5.10. One-way ANOVA results of strength index analysis before and after stimulation for each of five frequency band in BP-responders.

Band	ANOVA results		mean ± std	
	F-value	p-value	before	after
delta	1.143	0.285	0.315 ± 0.014	0.338 ± 0.015
theta	2.849	0.092	o.335 ± 0.015	0.300 ± 0.013
alpha	2.796	0.095	0.2596 ± 0.0098	0.2362 ± 0.0099
beta	13.725	0.000	0.0513 ± 0.0031	0.0699 ± 0.0038
gamma	9.433	0.002	0.1152 ± 0.0054	0.1400 ± 0.0059

Table S5. One-way ANOVA results of degree index analysis in consecutive sessions for MDD-responders and BP-responders. The consecutive ANOVA parameters in one column refer to differences between 1st and 10th, 1st and 20th, 10th and 20th session. The critical value of F is equal to -2.920.

Group	ANOVA results		mean ± std		
	F-value	p-value	1st	10th	20th
MDD-responders	56.490, 86.195, 4.494	0.000, 0.000, 0.034	3.398 ± 0.086	0.40 ± 0.10	4.72 ± 0.11
BP-responders	6.207, 41.097, 16.650	0.013, 0.000, 0.000	3.26 ± 0.090	3.585 ± 0.095	4.19 ± 0.11

Table S6. One-way ANOVA results of strength index analysis in consecutive sessions for MDD-responders and BP-responders. The consecutive ANOVA parameters in one column refer to differences between 1st and 10th, 1st and 20th, 10th and 20th session. The critical value of F is equal to -2.920.

Group	ANOVA results		mean ± std		
	F-value	p-value	1st	10th	20th
MDD-responders	37.335, 53.730, 1.718	0.000, 0.000, 0.190	0.2255 ± 0.0061	0.2824 ± 0.0069	0.2956 ± 0.0073
BP-responders	37.335, 53.730, 1.718	0.353, 0.000, 0.000	0.2188 ± 0.0065	0.2274 ± 0.0064	0.2733 ± 0.0078

Table S7. One-way ANOVA results of strength index analysis in consecutive sessions for MDD-responders. The consecutive ANOVA parameters in one column refer to differences between 1st and 10th, 1st and 20th, 10th and 20th session. The critical value of F is equal to 6.944.

Group	ANOVA results		mean ± std		
	F-value	p-value	1st	10th	20th
delta	44.001, 36.870, 0.280	0.000, 0.000, 0.597	0.367 ± 0.018	0.547 ± 0.020	0.532 ± 0.020
theta	21.217, 8.676, 2.710	0.000, 0.003, 0.100	0.250 ± 0.013	0.351 ± 0.016	0.313 ± 0.016
alpha	12.774, 10.678, 42.699	0.000, 0.001, 0.000	0.326 ± 0.014	0.258 ± 0.012	0.401 ± 0.017
beta	18.684, 13.300, 0.387	0.000, 0.000, 0.534	0.0409 ± 0.0030	0.0613 ± 0.0036	0.0582 ± 0.0036
gamma	16.809, 7.012, 2.724	0.000, 0.008, 0.099	0.1430 ± 0.0082	0.1941 ± 0.0093	0.1737 ± 0.0081

Table S8. One-way ANOVA results of strength index analysis in consecutive sessions for BP-responders. The consecutive ANOVA parameters in one column refer to differences between 1st and 10th, 1st and 20th, 10th and 20th session. The critical value of F is equal to 6.944.

Group	ANOVA results		mean ± std		
	F-value	p-value	1st	10th	20th
delta	44.001, 36.870, 0.280	0.000, 0.000, 0.597	0.367 ± 0.018	0.547 ± 0.020	0.532 ± 0.020
theta	21.217, 8.676, 2.710	0.000, 0.003, 0.100	0.250 ± 0.013	0.351 ± 0.016	0.313 ± 0.016
alpha	12.774, 10.678, 42.699	0.000, 0.001, 0.000	0.326 ± 0.014	0.258 ± 0.012	0.401 ± 0.017
beta	18.684, 13.300, 0.387	0.000, 0.000, 0.534	0.0409 ± 0.0030	0.0613 ± 0.0036	0.0582 ± 0.0036
gamma	16.809, 7.012, 2.724	0.000, 0.008, 0.099	0.1430 ± 0.0082	0.1941 ± 0.0093	0.1737 ± 0.0081

Table S9. One-way ANOVA results of strength index analysis in consecutive sessions for MDD-nonresponders. The consecutive ANOVA parameters in one column refer to differences between 1th, 10th and 1st, 20th, 10th and 20th session. The critical value of F is equal to 6.944.

Group	ANOVA results		mean ± std		
	F-value	p-value	1st	10st	20st
delta	1.942, 10.077, 21.296	0.165, 0.002, 0.000	11.54 ± 0.58	12.68 ± 0.57	9.02 ± 0.54
theta	24.422, 39.657, 2.172	0.000, 0.000, 0.142	5.89 ± 0.44	9.16 ± 0.48	10.21 ± 0.51
alpha	0.002, 0.041, 0.06	0.962, 0.839, 0.804	1.57 ± 0.22	1.56 ± 0.23	1.63 ± 0.21
beta	13.928, 0.234, 17.528	0.000, 0.629, 0.000	1.27 ± 0.16	2.35 ± 0.23	1.15 ± 0.15
gamma	1.140, 57.039, 54.451	0.287, 0.000, 0.000	3.79 ± 0.37	3.27 ± 0.31	0.73 ± 0.14

Table S10. One-way ANOVA results of strength index analysis in consecutive sessions for BP-nonresponders. The consecutive ANOVA parameters in one column refer to differences between 1st and 10th, 1st and 20th, 10th and 20th session. The critical value of F is equal to 6.944.

Group	ANOVA results		mean ± std		
	F-value	p-value	1st	10th	20th
delta	25.216, 24.480, 0.010	0.000, 0.000, 0.921	5.88 ± 0.29	3.92 ± 0.25	3.95 ± 0.25
theta	14.237, 4.235, 3.276	0.000, 0.040, 0.071	3.04 ± 0.22	2.00 ± 0.15	2.44 ± 0.18
alpha	65.575, 9.935, 22.147	0.000, 0.002, 0.000	4.33 ± 0.17	2.54 ± 0.13	3.56 ± 0.16
beta	5.108, 0.046, 3.490	0.024, 0.830, 0.062	1.27 ± 0.10	0.92 ± 0.11	1.24 ± 0.12
gamma	4.293, 2.966, 0.186	0.039, 0.085, 0.666	3.08 ± 0.18	3.70 ± 0.23	3.56 ± 0.20

Table S11. One-way ANOVA results of degree index analysis for MDD-responders and MDD-nonresponders. The critical value of F is equal to -161.448.

Condition	ANOVA results		mean ± std	
	F-value	p-value	BP-responders	BP-nonresponders
Before	23.174	0.000	3.787 ± 0.093	4.49 ± 0.11
After	4.504	0.034	4.006 ± 0.094	4.32 ± 0.11

Table S12. One-way ANOVA results of strength index analysis differences between MDD-responders and MDD-nonresponders groups before and after stimulation in different EEG bands. The critical value of F is equal to 7.709.

Band	ANOVA results		mean ± std	
	F-value	p-value	before	after
			**Before**	
delta	33.055	0.049	5.48 ± 0.26	7.89 ± 0.32
theta	8.895	0.003	3.63 ± 0.20	4.56 ± 0.23
alpha	39.261	0.000	4.71 ± 0.18	3.15 ± 0.16
beta	0.320	0.572	1.412 ± 0.096	1.337 ± 0.092
gamma	7.677	0.000	3.69 ± 0.21	5.52 ± 0.26
			**After**	
delta	38.268	0.000	6.16 ± 0.24	8.76 ± 0.34
theta	0.001	0.970	4.34 ± 0.22	4.35 ± 0.23
alpha	3.913	0.048	3.15 ± 0.15	2.70 ± 0.16
beta	3.642	0.057	1.488 ± 0.095	1.76 ± 0.10
gamma	6.144	0.013	4.89 ± 0.24	4.03± 0.24

Table S13. One-way ANOVA results of degree index analysis for BP-responders and BP-nonresponders. The critical value of F is equal to 161.448.

Condition	ANOVA results		mean ± std	
	F-value	p-value	BP-responders	BP-nonresponders
Before	3.310	0.069	3.357 ± 0.074	3.140 ± 0.093
After	25.732	0.000	3.614 ± 0.079	2.989 ± 0.092

Table S14. One-way ANOVA results of degree index analysis differences between BP-responders and BP-nonresponders groups before and after stimulation in different EEG bands. The critical value of   F is equal to 7.709.

Band	ANOVA results		mean ± std	
	F-value	p-value	before	after
			**Before**	
delta	7.380	0.007	0.315 ± 0.014	0.384 ±0.021
theta	41.651	0.000	0.335 ± 0.015	0.189 ±0.014
alpha	0.059	0.809	0.2600 ± 0.0098	0.256 ±0.011
beta	6.439	0.011	0.051 ± 0.0031	0.03866 ± 0.0039
gamma	0.868	0.352	0.1152 ± 0.0054	0.1234 ± 0.0070
			**After**	
delta	3.870	0.049	0.338 ± 0.015	0.389 ± 0.021
theta	25.888	0.000	0.300 ± 0.013	0.195 ± 0.014
alpha	3.388	0.066	0.2362 ± 0.0099	0.2094 ±0.0098
beta	22.025	0.000	0.06987 ± 0.0038	0.0435 ± 0.0041
gamma	7.677	0.006	0.1399 ± 0.0059	0.1136 ± 0.0074

Table S15. One-way ANOVA results of degree index comparison between MDD-responders and BP-responders as well as between MDD-nonresponders and BP-nonresponders before and after rTMS stimulation. The critical value of F is equal to 161.448.

Band	ANOVA results		mean ± std	
	F-value	p-value	MDD group	BP group
MDD/BP responders				
Before	12.972	0.000	3.787 ± 0.093	3.357 ± 0.074
After	9.892	0.002	4.005 ± 0.094	3.514 ± 0.079
MDD/BP nonresponders				
Before	85.730	0.000	4.49 ± 0.11	3.140 ± 0.093
After	82.475	0.000	4.32 ± 0.011	2.989 ± 0.092

Table S16. One-way ANOVA results of degree index analysis differences between MDD-responders and BP-responders groups before and after stimulation in different EEG bands. The critical value of F is equal to 7.709.

Band	ANOVA results		mean ± std	
	F-value	p-value	BP-responders	MDD-responders
Before				
delta	21.145	0.000	0.427 ± 0.20	0.315 ±0.014
theta	6.961	0.010	0.275 ± 0.015	0.335 ±0.015
alpha	29.840	0.000	0.352 ± 0.014	0.2597±0.0098
beta	0.009	0.926	0.05181 ± 0.0035	0.05137 ± 0.0031
gamma	11.337	0.001	0.1475 ± 0.0084	0.1152 ± 0.0054
After				
delta	36.087	0.000	0.486 ± 0.018	0.338 ± 0.015
theta	1.410	0.235	0.326 ± 0.016	0.300 ± 0.013
alpha	0.078	0.780	0.232 ± 0.012	0.2362 ±0.0099
beta	12.813	0.000	0.0504 ± 0.0032	0.06987 ± 0.0038
gamma	22.470	0.000	0.1897 ± 0.0091	0.1400 ± 0.0059

Table S17. One-way ANOVA results of degree index analysis differences between MDD-nonresponders and BP-nonresponders groups before and after stimulation in different EEG bands. The critical value of F is equal to 7.709.

Band	ANOVA results		mean ± std	
	F-value	p-value	MDD-nonresponders	BP-nonresponders
Before				
delta	52.620	0.000	7.89 ± 0.32	4.790 ±0.27
theta	48.044	0.000	4.56 ± 0.23	2.45 ±0.19
alpha	8.357	0.004	3.15 ± 0.16	3.84±0.17
beta	4.073	0.44	1.337 ± 0.092	1.05 ± 0.11
gamma	32.478	0.000	5.52 ± 0.26	3.58 ± 0.20
After				
delta	71.609	0.000	8.76 ± 0.34	5.01 ± 0.28
theta	34.125	0.000	4.36 ± 0.23	2.58 ± 0.19
alpha	2.222	0.136	2.70 ± 0.16	3.03 ±0.14
beta	15.708	0.000	1.76 ± 0.10	1.14 ± 0.11
gamma	6.908	0.009	4.04 ± 0.24	3.19 ± 0.21
